# The Labor Supply of Full-Time and Part-Time Pharmacists in the U.S.

**DOI:** 10.3390/pharmacy14040106

**Published:** 2026-07-11

**Authors:** Ioana Popovici, Manuel J. Carvajal, Rawan Alkhamisi

**Affiliations:** Department of Sociobehavioral and Administrative Pharmacy, Barry and Judy Silverman College of Pharmacy, Nova Southeastern University, 3200 South University Drive, Fort Lauderdale, FL 33328, USA; cmanuel@nova.edu (M.J.C.); ra1606@mynsu.nova.edu (R.A.)

**Keywords:** earnings, gender, human capital, income gaps, job-related preferences, labor input, part-time employment, pharmacist workforce

## Abstract

The U.S. pharmacist workforce has undergone significant growth and demographic changes, particularly increased female participation and part-time employment. However, limited evidence exists on how labor supply behavior varies by gender and employment status. This study used a nationally representative sample of 12,064 pharmacists from the 2019–2022 American Community Survey (ACS) to examine heterogeneity in labor supply decisions. Ordinary Least Squares (OLS) regression models were estimated separately by employment status (full-time versus part-time) and gender. The dependent variable was the number of hours worked. Independent variables included wage rate, age, marital status, number of children, and race/ethnicity. Findings showed that women were more than twice as likely to work part-time as men and minority pharmacists were largely underrepresented in the study sample relative to nonminority pharmacists. Gender gaps in wages, hours worked, and demographic and family characteristics also differed markedly between full-time and part-time pharmacists. Finally, findings lent support to the contention than women are more likely to work fewer hours than men as they are expected to spend more hours at home raising children. Understanding how individual characteristics shape pharmacists’ labor supply, and how these relationships differ by gender and employment status, is essential for workforce planning and healthcare human resources policy.

## 1. Introduction

Pharmacy is one of the fastest growing healthcare professions in the United States [1]. Between 2014 and 2022, pharmacist employment expanded by 25.5%, with notable gains in nontraditional sectors such as ambulatory care, health informatics, and general merchandise retail [2,3]. This expansion has been accompanied by a rising share of women in the profession; female composition grew from about 60% in 2022 [2] to 68.1% in 2024 [4]. Such increasing participation of women is relevant for workforce dynamics, as female pharmacists historically have supplied on average fewer hours than their male counterparts, largely due to child-rearing and household responsibilities [5].

Despite women constituting most of the pharmacist workforce, gender earnings disparities are well documented [6]. One study found a gender earnings gap of up to 18.6% in favor of male pharmacists even after controlling for human capital and job-related characteristics, suggesting that gender independently influences earnings determination [7]. Other studies have similarly documented persistent wage differentials in the profession [8].

Understanding how individual characteristics shape pharmacists’ labor supply, and whether these relationships differ by gender and employment status, is essential for workforce planning and healthcare human resources policy. The present study addressed this gap by estimating labor supply functions for U.S. pharmacists, separately by employment status (full-time versus part-time) and gender. Specifically, this study sought to (1) formulate, estimate, and test parameters of a labor supply function separately, within each gender, for U.S. full-time and part-time pharmacists; (2) compare the empirical findings between employment-status groups and between genders within each employment-status group; and (3) evaluate the evidence for policy implications.

## 2. Methods

The study employed ordinary least-squares (OLS) regression to estimate a model of pharmacists’ labor supply as a function of wage rates and selected human capital, family, and demographic characteristics. Conceptually, the model estimated how pharmacists’ annual hours worked vary with wage rates, age, family characteristics, and demographic factors, separately for men and women working full-time and part-time. In the classical labor–leisure framework, individuals choose hours of work by maximizing utility subject to a budget constraint, where wages determine the opportunity cost of leisure and non-labor income shapes overall labor supply [9]. Within this context, wage changes generate both substitution effects, which tend to increase hours worked, and income effects, which may reduce hours worked as individuals reach desired income levels. The inclusion of age in the model is consistent with human capital theory and life-cycle labor supply behavior, whereby accumulated experience and proximity to retirement influence labor market participation and hours supplied over time. Family and demographic characteristics further capture constraints and preferences that shift the feasible set of labor–leisure choices. Together, these theoretical perspectives provide a framework for interpreting the estimated associations between wages, individual characteristics, and pharmacists’ labor supply decisions.

The sample was divided into two employment-status groups: full-time pharmacists, who worked at least 32 h per week in the past year, and part-time pharmacists, who worked less than 32 h per week but at least 100 h in the past year. This definition was selected because it is consistent with prior studies examining pharmacists and other healthcare professionals [10,11]. The selected cutoff is consistent with, and slightly more restrictive than, the 30 h criterion used by the Internal Revenue Service and the Affordable Care Act to define full-time employment for purposes of employer-sponsored health insurance coverage [12,13].

### 2.1. The Model and Its Variables

The same specification was applied to male and female pharmacists within each employment-status group, a total of four equations. The average number of annual hours worked was formulated as follows:

H_ijk_ = α_ij_ + W_hijk_β_hij_ + A_hijk_λ_hij_ + X_hijk_γ_hij_ + Z_hijk_ϕ_hij_ + u_ijk_

where

H_ijk_ was a vector of values of the number of hours worked in the past year reported by the *k*th pharmacist of the *j*th gender within the *i*th employment-status group;

W_hijk_ was a matrix of values (*h* = 1, 2) of the linear and quadratic terms of the hourly wage rate, in dollars, reportedly earned by the *k*th pharmacist of the *j*th gender within the *i*th employment-status group;

A_hijk_ was a matrix of values (*h* = 1, 2) of the linear and quadratic terms of age reported by the *k*th pharmacist of the *j*th gender within the *i*th employment-status group;

X_hijk_ was a matrix of values of family characteristics, including being married (*h* = 1) and number of children in the household (*h* = 2), reported by the *k*th pharmacist of the *j*th gender within the *i*th employment-status group;

Z_hijk_ was a matrix of values of demographic characteristics, including being Black/African American (*h* = 1) and Hispanic (*h* = 2), reported by the *k*th pharmacist of the *j*th gender within the *i*th employment-status group;

u_ijk_ was a vector of normally and independently distributed stochastic disturbance terms, with mean zero and variance σ_ij_^2^, for the *k*th pharmacist of the *j*th gender within the *i*th employment-status group;

α_ij_ was the least-squares constant term estimated for the *j*th gender within the *i*th employment-status group; and

β_hij_, λ_hij_, γ_hij_, and ϕ_hij_ were vectors of two parameters, one parameter per covariate (plus transformation, if any) within their respective matrices, being estimated for the *j*th gender of the *i*th employment-status group using ordinary least-squares; and

where

*h* = 1, 2 for the parameters within each matrix;

*j* = 1 for male pharmacists and *j* = 2 for female pharmacists;

*i* = 1 for full-time pharmacists and *i* = 2 for part-time pharmacists; and

*k* = 1, …, n_ij_ for the number of pharmacists of the *j*th gender within the *i*th employment-status group.

The four equations included identical sets of covariates to enable direct comparison of the sign, magnitude, and statistical significance of coefficients affecting labor supply, holding all other factors constant. This approach minimized differences that might have been attributed to inter-covariate interactions. Alternative specifications, such as models capturing gender and/or employment status through dichotomous variables, were not pursued as they imposed the restrictive and likely unrealistic assumption that responses to covariates are homogeneous across pharmacist groups [7]. In fact, disparities in response, if they existed, were the type of empirical evidence sought by this study. All analyses used the ACS sampling weights to provide unbiased and representative estimates for the target populations.

Traditionally, the wage rate is the main covariate of pharmacists’ labor supply functions [14]. Hourly wage rates were derived by dividing respondents’ annual pre-tax wage and salary income by the product of the number of weeks worked during the previous year times the usual number of hours worked per week. The resulting wage measure was then adjusted to 2022 dollars using the Consumer Price Index (CPI). Pharmacists who worked fewer than 100 h during the previous year and those with calculated hourly wage rates below $10 per hour were excluded. No additional winsorization or trimming of wage observations was performed. In this model the wage rate was specified with both linear and quadratic components to allow for potential nonlinearity in the labor supply function. This captured the possibility of a “bend” reflecting two opposing forces: the substitution effect, whereby higher wages encourage pharmacists to substitute leisure for work as the return to labor increases, and the income effect, whereby higher earnings enable pharmacists to reduce the number of hours worked while still achieving preferred consumption–leisure combinations. Thus, the linear coefficients were expected to be positive, and the quadratic coefficients were expected to be negative.

Age was included in the model as a proxy for labor experience, a human-capital construct. It also appeared with both linear and quadratic components. The number of working hours was hypothesized to increase with age at a decreasing rate—more hours beyond the family formation years, especially for women, but relatively fewer hours as pharmacists approach retirement. Following this logic, the linear coefficients were expected to be positive, and the quadratic coefficients were expected to be negative.

The two family characteristics included in the model were a dichotomous covariate for current marital status (a value of one if the pharmacist was married, a value of zero otherwise) and the number of children in the household. Both variables have been shown to reduce labor supply among women, while in some cases increasing the number of hours worked by men [15,16,17]. The needs and demands of household duties and parental supervision are likely to differ between genders and employment-status groups [18]; consequently, the influence of both covariates on supplied labor was expected to be dissimilar across groups.

Several studies have found that both race and ethnicity are associated with labor market outcomes [19,20,21]. Within pharmacy, racial and ethnic minority practitioners have exhibited different labor supply responses to human-capital variables when compared with nonminority pharmacists [7,22,23]. In this study, two binary covariates were included to indicate whether a pharmacist self-identified as Black/African American and of Hispanic origin. No hypotheses regarding the coefficients were formulated.

### 2.2. Endogeneity Concerns

The estimation of labor supply and/or demand equations using ordinary least-squares often is challenged on the grounds of simultaneity bias. Simultaneity arises when the quantity of labor supplied, the dependent variable, and the wage rate, its primary covariate, are jointly determined, leading to a correlation between the wage variable and the stochastic error term; such correlation is not consistent with the unbiasedness assumption of ordinary least-squares. According to this argument, most applicable to standard market models, the equilibrium quantity of labor supplied reflects the aggregate work effort over time; changes in the wage rate may lead pharmacists to adjust hours worked through substitution and income effects; these adjustments, in turn, can influence subsequent equilibrium wages and hours in later periods.

The methodological scope here was different. The focus was on the behavior of individual pharmacists through observations from cross-sectional data. The wage rate was hypothesized to influence the amount of labor supplied, but labor supplied was not expected to have a substantial contemporaneous effect on the wage rate. To the extent that such an effect exists, it would likely emerge in a subsequent period not captured by the model. Moreover, any resulting bias would be expected to affect the equations of pharmacists from both genders and both employment-status groups, thereby largely canceling out when comparing the estimated least-squares coefficients for each covariate. To dispel all doubt, the correlation coefficients between the wage rate and the residuals for each of the four equations were calculated and tested for statistical significance.

### 2.3. Data

The 2019–2022 American Community Survey (ACS), conducted by the U.S. Census Bureau, was the data source used for this study. Every year the ACS randomly gathers data on economic, social, and labor force characteristics from over 3 million households throughout the 50 states, Washington, D.C., and Puerto Rico. Pharmacists were selected from the global survey using the harmonized U.S. Census Bureau’s 2010 ACS occupation classification scheme. To reduce the influence of outliers, the sample was limited to pharmacists who worked at least 100 h and earned an average hourly wage rate of at least $10.00 per hour during the previous year; these parameters were set to exclude outliers. The sample consisted of 12,064 practitioners (4667 men and 7397 women) between the ages of 25 and 64 years working in the U.S. Their incomes were adjusted for inflation to 2022 dollars using the Consumer Price Index (CPI). The research endeavor was supported solely by internal university funds and Institutional Review Board’s assessment was not needed because the data were deidentified and publicly accessible.

### 2.4. Results

Empirical evidence revealed that 14.5% of pharmacists in the sample worked part time (e.g., less than 32 h per week in the past year). This practice was much more common among women (18.6%) than among men (8.2%).

### 2.5. Employment-Status Group Comparisons

Table 1 reports the means and standard deviations of labor supply and its hypothesized determinants by gender for full-time and part-time pharmacists. Significant differences between genders and between employment-status groups were established using the t statistic with uneven numbers of observations. On average, full-time male pharmacists worked longer hours than female pharmacists, but the opposite was true for part-time pharmacists. Full-time male pharmacists also earned higher wage rates than their female counterparts, but no gender difference was detected for part-time pharmacists; no significant differences in wage rates were observed between full-time and part-time pharmacists of the same gender either.

Within full-time pharmacists, men were older than women and women who worked part time were older than women who worked full time. Proportionately more male than female pharmacists who worked full time reported being married, as did proportionately more women than men who worked part time. The same pattern was observed for the number of children present in the household—higher for men than women who worked full time but higher for women than men who worked part time. Similarly, there were proportionately more Black/African American pharmacists among women than men who worked full time but more among men than women who worked part time, and the prevalence of Black/African Americans was higher for full-time than part-time female pharmacists. The only significant difference for Hispanic ethnicity was a greater proportion for part-time men than women.

### 2.6. Estimated Equations

Table 2 reports the estimated least-squares coefficients, standard errors, and significance level for all covariates. Each covariate coefficient was significant for at least one of the employment-status groups and the correlation coefficients between the wage rate and the residuals lacked significance (ρ ≤ 0.10) in all four equations. The F-statistics indicated that all four models were jointly significant, and the adjusted R^2^ values were comparable across equations. The wage-rate coefficients were not statistically significant for male participants working part time but were significant for the other three groups.

The linear coefficients were negative, and the quadratic coefficients were positive, which was the opposite of what had been hypothesized. The effect of this covariate was examined further through the estimation of labor supply elasticities (see Table 3). Evaluated at the sample means, these elasticities represent the percentage change in the quantity of labor supplied associated with an infinitesimal percentage change in the covariate; specifically, the estimated wage-rate elasticity for full-time male pharmacists indicated that a 10% rise in the wage rate is associated with a 1.0% decrease in hours worked. Similar interpretations apply to the remaining employment-status and gender groups.

The coefficients for age behaved as expected—linear terms were positive and quadratic terms were negative, all statistically significant. At the means of the variables, an additional year of age brought about 3.3 more hours worked per year for full-time male pharmacists, 2.9 additional hours for full-time female pharmacists, 8.6 additional hours for part-time male pharmacists, and 8.2 additional hours for part-time female pharmacists. This covariate was inelastic for all four groups, more so for full-time than part-time pharmacists (see Table 3).

The only marriage coefficient that turned out statistically significant was for male pharmacists who worked full time; on average, these pharmacists worked 52.7 more hours per year than their nonmarried counterparts. The coefficients for presence of children in the household were negative for female pharmacists, as expected, but lacked statistical significance for male pharmacists. The reduction in hours observed for women due to the presence of children was proportionately greater for part-time than full-time practitioners.

The coefficients for Black/African American male pharmacists were negative and significant, but the coefficients for Black/African American women pharmacists were not significant. For men, the reduction in hours compared to their non-Black/African American counterparts was over four times greater for part-time than full-time pharmacists, and it was even greater percentagewise. The pattern for Hispanic pharmacists was similar—negative and significant for men but not for women, and a greater effect for part-time than full-time pharmacists.

## 3. Discussion

The statistical significance of the least-squares coefficient estimates and the F ratios suggests that the model developed and tested in this article was moderately successful in describing the amount of labor supplied by full-time and part-time pharmacists in the U.S. The relatively low R^2^ values and large constant terms suggest that the addition of other covariates such as practice setting, pharmacist role, burnout and work satisfaction, and other workplace characteristics, as well as spouse employment and educational debt, not included here, might increase goodness of fit. Accordingly, the models should be viewed as providing a partial rather than a comprehensive explanation of pharmacists’ labor supply behavior. The absence of statistical significance in the correlation coefficients between the wage rate and the residuals provided some evidence against contemporaneous simultaneity. Nevertheless, this should be interpreted cautiously, as this does not constitute a formal econometric test for endogeneity. Finally, other sources of endogeneity, including omitted variables, occupational sorting, and measurement error, cannot be ruled out. Consequently, the estimated coefficients should be interpreted as associations rather than causal effects.

The empirical evidence lends itself to four generalizations. First, gender played a decisive role in the observed behavior of the variables analyzed in this study. Not only was the practice of part-time work over twice as high among women (18.6%) as among men (8.2%), but within full-time pharmacists the average wage rate and age of male practitioners were higher than those of female practitioners. In addition, the relationship between genders for the other variables was the opposite for full-time and part-time pharmacists. Men who worked full time worked on average more hours than women who worked full time, were more likely to be married, had more children in the household, and were less likely to be Black/African American, whereas women who worked part time worked more hours than men who worked part time, were more likely to be married, had more children in the household, and were less likely to be Black/African American. Female pharmacists who worked part time also were less likely to be Hispanic than their male counterparts. The older age of female part-time pharmacists relative to their full-time peers supports the contention that women choose to retire gradually, working progressively fewer hours as they grow older. Observed gender differences may reflect different household responsibilities and caregiving but also broader institutional and labor market factors, including workplace flexibility, occupational segmentation, and structural constraints within healthcare employment.

Second, and most puzzling, is that the estimated signs of the significant wage-rate coefficients ran counter to expectations: the linear terms were negative and the quadratic terms positive, yielding negative elasticities. Although small in magnitude, consistently with positions along the backward bending portion of the labor supply curve, these findings should be interpreted with caution. Given the cross-sectional nature of the data and the limited set of covariates available, the estimated associations do not necessarily represent causal labor supply responses to changes in wages. Rather, they may reflect unobserved differences across pharmacists and practice settings. Several factors may contribute to the observed negative relationship between wages and hours worked. First, compensation structures vary substantially across pharmacy settings, with some pharmacists receiving salaried compensation while others paid on an hourly basis. Second, they may capture job sorting and/or measurement issues, whereby higher-wage pharmacists are more likely to hold hospital or administrative positions with fewer hours, while lower-wage pharmacists are concentrated in retail settings with longer shifts and greater total hours, generating an apparent negative relationship between wages and hours worked. In addition, regional differences in wages and costs of living, selection into retail versus institutional practice settings, and labor market disruptions associated with the COVID-19 pandemic may have influenced both wages and hours worked. Thus, the negative elasticities observed in this study may reflect occupational sorting, compensation heterogeneity, or omitted-variable bias rather than true backward-bending labor supply behavior. Yet, the findings of three negative labor supply elasticities derived from statistically significant estimates warrant further investigation. They suggest that the relationship between wages and hours worked among pharmacists may be more complex than predicted by standard labor supply models. In contrast, the lack of significance for the wage-rate coefficients among male pharmacists working part time suggests that their limited hours are less strongly associated with wage variation and may instead reflect institutional constraints or preferences regarding work schedules and leisure.

The third generalization is that all four age elasticities of labor supply were positive, and the values for part-time pharmacists exceeded those of full-time pharmacists of the same gender. This implies that part-time practitioners worked proportionately more hours with each additional year than full-time practitioners. The presence of children in the household had a negative impact on women’s number of hours worked in both employment-status groups, affecting part-time workers slightly more than full-time workers. Nevertheless, no appreciable effect was observed among male pharmacists; this finding lends support to the contention that relatively more women than men work fewer hours in the labor force because they are expected to work more hours at home raising children [15,16,17].

Finally, the empirical evidence revealed that minority pharmacists are largely underrepresented in the study sample, consistent with national workforce data indicating that several racial and ethnic minority groups remain underrepresented in the U.S. pharmacy profession [24,25]. Fewer than one out of every 20 participants were Black/African American or Hispanic. Male practitioners belonging to either racial/ethnic group worked significantly fewer hours than nonminority male practitioners, especially if they were classified as part-time workers. Women minority pharmacists did not seem to be affected in terms of the number of hours worked.

## 4. Limitations

The findings of this article should be interpreted in light of the study’s specific methodological framework and its associated limitations. Accordingly, several limitations ought to be identified. The structure of the equations was restricted by the chosen covariates; if additional covariates had been introduced, the goodness of fit might have improved. The study was focused on self-reported data, which raises validity and reliability concerns; responses might have been subject to emotional sways by participating pharmacists. Moreover, there was no way of corroborating the accuracy of responses or even asserting that the responses were provided by the intended practitioners.

Another limitation is that the scope focused on a specific period, namely 2019–2022. Thus, its methodology was not adequate to ascertain if the labor supplied by both genders within full-time and part-time pharmacists change over time, or how the changes are affected by the steady increase in the number of pharmacy school graduates. Moreover, because the study period encompassed the COVID-19 pandemic years, the observed labor force behavior of pharmacists may not be fully representative of long-run patterns. The pandemic may have had a substantial impact on pharmacists’ labor supply behavior. Pandemic-related factors, including changes in working hours, temporary labor market disruptions, staffing shortages, burnout, caregiving responsibilities, and transitions between full-time and part-time employment, may have influenced the observed patterns. For example, during the pandemic, the demand for pharmacists surged [26] and some pharmacists worked extended hours to meet the need [27]. Some of the relationships identified in this study may reflect conditions specific to the pandemic period and therefore may not fully represent long-term workforce behavior.

Another limitation is that adjusting income values to 2022 dollars using the CPI is somewhat arbitrary and may obscure meaningful within-period variation. In particular, it does not capture potential year-to-year changes in pharmacists’ incomes across the 2019, 2020, and 2021 sample years. Furthermore, the reported levels of income and wage rates did not take into account spatial variations in cost of living and taxes, which are essential in configuring real income; oftentimes real wage rates influence socioeconomic behavior more accurately than nominal wage rates. Finally, several factors may contribute to the observed negative relationship between wages and hours worked. These include differences in compensation structures, salaried versus hourly employment arrangements, managerial or administrative responsibilities, and selection into different pharmacy sectors. Since these factors were not explicitly incorporated into the present model specification, the estimated wage coefficients may partially capture their influences rather than pure labor supply responses. More generally, although the analysis addressed concerns regarding contemporaneous simultaneity, other sources of endogeneity, including omitted variables, measurement error, occupational self-selection, and unobserved productivity differences, cannot be ruled out. Consequently, the estimated coefficients should be interpreted as statistical associations rather than causal effects. Future research employing alternative identification strategies may provide additional insight into the causal relationship between wages and labor supplied by pharmacists.

## 5. Conclusions

Despite its limitations, this study has provided a useful framework for examining the nature of the labor supplied by full-time and part-time pharmacists in the U.S. It has established that both employment-status groups and both genders within each group behave differently in terms of labor supply and respond dissimilarly to identical socioeconomic stimuli. It also has raised questions pertaining to labor supply elasticities that need be addressed.

Future research should incorporate a longitudinal perspective to better capture changes in the role and importance of labor supply over time. Hopefully, the theoretical context, methods, and empirical findings of this study may serve as a guide to further endeavors.

## Figures and Tables

**Table 1:** Means and standard deviations (in parentheses) of variables hypothesized to influence pharmacists’ labor supply in the U.S., by workforce-status group and gender.

Variable	Means and Standard Deviations (in Parentheses)			
	Full-Time Pharmacists		Part-Time Pharmacists	
	Men	Women	Men	Women
Number of observations	4286	6024	381	1373
Work per year (hours)	2147.0 ^ay^	2084.4 ^ay^	902.0 ^az^	968.3 ^az^
	(440.1)	(423.0)	(485.3)	(460.9)
Wage rate ($/hour)	69.1 ^y^	64.9 ^y^	64.9	67.7
	(50.6)	(52.3)	(71.5)	(51.4)
Age (years)	42.7 ^y^	40.9 ^ay^	43.8	43.4 ^a^
	(11.0)	(10.4)	(14.7)	(11.1)
Married (%)	0.724 ^ay^	0.687 ^ay^	0.577 ^ay^	0.785 ^ay^
	(0.447)	(0.464)	(0.495)	(0.411)
Children in the household (#)	1.03 ^ay^	0.93 ^ay^	0.46 ^ay^	1.17 ^ay^
	(1.21)	(1.07)	(0.85)	(1.20)
Black/African American (%)	0.045 ^z^	0.056 ^az^	0.060 ^z^	0.029 ^az^
	(0.207)	(0.230)	(0.238)	(0.168)
Hispanic (%)	0.044	0.043	0.063 ^z^	0.034 ^z^
	(0.206)	(0.203)	(0.243)	(0.182)

^a^ Significant difference between employment-status groups within the same gender (ρ ≤ 0.01). ^y^ Significant difference between genders within the same employment-status group (ρ ≤ 0.01). ^z^ Significant difference between genders within the same employment-status group (ρ ≤ 0.05).

**Table 2:** Estimated least-squares coefficients, their standard errors (in parentheses), and levels of significance.

Covariate	Term	Estimated Least-Squares Coefficients, Standard Errors, and Levels of Significance			
		Full-Time Pharmacists (*i* = 1)		Part-Time Pharmacists (*i* = 2)	
		Men (*j* = 1)	Women (*j* = 2)	Men (*j* = 1)	Women (*j* = 2)
Constant term	α	1495.14	1698.89	−1222.72	−1159.96
Wage rate	β_1_	−3.342769 *	−4.674889 *	−1.159886	−1.944802 *
		(0.269915)	(0.219821)	(0.805498)	(0.438756)
Wage rate squared	β_2_	0.001267 *	0.002235 *	0.000036	0.000236
		(0.000313)	(0.000218)	(0.001268)	(0.000785)
Age	λ_1_	39.215315 *	31.968944 *	104.096515 *	101.997283 *
		(6.052270)	(4.787642)	(18.812531)	(11.164949)
Age squared	λ_2_	−0.420599 *	−0.355121 *	−1.090439 *	−1.080115 *
		(0.067917)	(0.054948)	(0.207245)	(0.126984)
Married	γ_1_	52.714148 *	23.518457	−24.300819	39.850644
		(16.924587)	(12.372135)	(55.086185)	(33.545758)
Number of children in the household	γ_2_	−3.556947	−24.827073 *	8.870043	−35.241097 *
		(6.681564)	(5.808404)	(32.263560)	(12.567735)
Black/African American	ϕ_1_	−66.395518 ^†^	−2.843989	−282.705179 *	−21.415943
		(25.977897)	(19.503232)	(84.650652)	(56.318254)
Hispanic	ϕ_2_	−85.288422 *	21.434771	−194.863393 ^†^	−91.657194
		(29.386183)	(24.388296)	(83.370950)	(64.481417)
*F* statistic		48.34 *	107.8 *	8.8 *	23.0 *
Adjusted R^2^		0.081	0.124	0.140	0.113

* Coefficient statistically significant (ρ ≤ 0.01). ^†^ Coefficient statistically significant (ρ ≤ 0.05).

**Table 3:** Labor supply elasticities, calculated at the means of the variables, derived from the values of the estimated coefficients.

Covariate	Estimated Labor Supply Elasticities			
	Full-Time Pharmacists		Part-Time Pharmacists	
	Men	Women	Men	Women
Wage rate	−0.102	−0.145	*	−0.134
Age	0.066	0.057	0.416	0.369

* Coefficients not statistically significant.

## Data Availability

The data presented in this study are available in IPUMS USA at https://usa.ipums.org/usa/, reference number [28]. These data were derived from the following resources available in the public domain: U.S. Census Bureau. American Community Survey. Retrieved 15 December 2025, from https://usa.ipums.org/usa/.
